# A New Order of General Practitioners

**Published:** 1893-08-19

**Authors:** 


### The Hospital

An Institutional Journal of

Science, Medicine, Nursing, and Philanthropy.

*For Hospitals, Asylums, Infirmaries, Dispensaries, Medical Practitioners, Students, Nurses, and the Charitable Public.*

Vol. XIV.—No. 360.] [August 19, 1893.

### A New Order of General Practitioners.

"She goes out every morning at about ten o'clock to make her first round, pretty much like a doctor, spending ten minutes or a quarter of an hour in each house. Helpless and lonely patients may detain her much longer, but if there are relations and friends her duty is to direct more than to undertake the actual nursing."

This little paragraph is copied unaltered from a published article on "The Parish Nurse." The article in question describes the present position and actual doings of a particular parish nurse and of parish nurses in general. The resemblance which this nurse bears to the parish doctor is striking, and is not to be mistaken. We see her leaving her house at ten in the morning neatly dressed and gloved. She sets out in an entirely business-like way "to make her first round." She has a good many "patients," so many that she cannot spend more than "ten minutes or a quarter of an hour in each house." In many cases it is her duty to "direct more than to undertake the actual nursing." Here almost everything is done which the doctor does, and done at the same time and in the same way exactly as he would do it. The same hour in the morning is chosen for the "first round"; the same time is devoted to each visit—"ten minutes or a quarter of an hour"; in many instances no actual work is done; it is the nurse's duty "to direct" rather than to do the "actual nursing." One thing and one thing only is lacking to make the description tally exactly; the nurse has not yet begun to order the physic. But that this is coming next in the order of development hardly any person "who knows" will venture for a moment to doubt.

Now, there are two classes of persons who are profoundly interested in this obvious development. The first are those who admit responsibility for the progress of medicine as a science and an art, and the second are those general practitioners of medicine who constitute the great body of the profession. Before proceeding to argue the question with these two classes we will cite a few additional and significant facts which are well known to experienced persons who are "in the swim."

The facts already noted show how the nurse is slowly but surely displacing the doctor in the cottage of the labourer and the home of the smaller tradesman. But that the nurse is also competing with the family physician to his serious injury among the rich and prosperous will be convincingly realised if the circumstances now to be recorded are borne in mind. Everybody has heard of home hospitals in London which have achieved public recognition. But how many "nursing homes" are there which are practically identical with home hospitals, and which compete with the general practitioner, but which are never publicly heard of? Of these latter private ventures we do not hesitate to say that there are scores in London alone, and an unknown number in the other large towns of the country. But what is the function of a home hospital, or of a private home where the wealthy sick are cared for? There is hardly any need to answer this question. Everybody knows that these private homes act as lion's providers for metropolitan specialists at the expense of general practitioners. The *modus operandi* is perfectly familiar. A wealthy patient of a private practitioner consults a specialist, probably in the first instance with the concurrence of his, or more commonly of her, family physician. The specialist counsels an operation or a period of residence under his care. A "nursing home" is selected, a nurse is duly installed, and the patient is at once placed under the sole and absolute direction of the specialist and the nurse. The family physician is "squeezed out" by the simplest and easiest process imaginable. "*Vale, vale, in eternum vale,*" he may now say to his patient, for in all probability he will never, in his capacity of doctor, set eyes upon her any more. The specialist will operate upon her; the nurse will attend her from day to day. At the end of a few weeks, if convalescence sets in, the specialist will order his patient to the seaside, and the nurse will duly accompany her. When recovery is well advanced the patient will return to her home; to her home, but not to her family doctor, for the nurse, will still be very frequently in charge, passing instruments, attending to diet, or to little aberrations in the symptoms, reporting to the specialist, and, under his directions, dealing entirely with every detail of the case.

Now we are not saying whether this condition of affairs is right or wrong; we are simply affirming that it exists; that it is spreading daily in every direction, and that it threatens the very existence of all classes of general practitioners, except the very few who, by the force of a strong personality, can command an all-round success equal to or better than the consultant or the specialist. This condition of things, we affirm, exists; and it is carefully fostered by a large number of the most prominent persons in the ranks of consultants and specialists, who are leaving no stone unturned in order that they may place nurses on a professional "register," which is to be comparable, as far as possible, with the "register" of medical practitioners. This is the point of our contention, not that it is wrong or right, for consultants and so-called "registered" nurses to squeeze out the general practitioner, but that it is the fact. It is the fact as surely as the agitation for Home Rule in Ireland is the fact, and if medical men have any practical capacity for their own business as a profession, they will recognise the fact and take it seriously to heart.

For our own part, the subject chiefly concerns us as a development in medicine, which cannot but have important bearings on the future of medicine and surgery as a science and an art. What will be the effect upon medical science if the great body of its practical bedside workers are to be women who have had only a nurse's training, acting under the direction of specialists and consultants, who must in most cases live at a distance? That is the real question at issue. That is the question which consultants and specialists themselves, if they were loyal to the highest ideals of medical progress, would ask themselves. It is also the question which the cultured general practitioner should ask himself; for the cultured practitioner of the family physician class has exactly the same right in the higher medicine, and the same loyalty to it, and responsibility for it, as the consultant and the specialist. Can the practitioner who has learnt to love medicine as the patriot loves his country, stand by in dull indifference while he sees his science and his art handed over to mere nurses, bedside workers, who know nothing of medicine but the plainest "rule of thumb?"

It is well to train nurses for nursing, and to give them their due place in the general scheme of medical and surgical practice. But when a deliberate attempt is made to place nurses on a "registered" level with "registered" medical men, and in practice to substitute the "family nurse" for the "family doctor" in the cottages of the poor and at the bedsides of the rich, then it is time for every man who has vested interests in medicine, and is loyal to the science and art of which he is a representative, to buckle on the armour of his sternest reason, and to demand whither it is that our so-called "leaders" are leading us. The watchword for every medical practitioner who is intelligently loyal to the highest ideals of medicine as a science and a cultured art must be this: Let nothing be done by consultant, specialist, or general practitioner, in the hospital or in the home, which will tend to convert the general practice of the medical art into a mere "rule of thumb," and to bury in a money-maker's oblivion the noble achievements of the fathers and pioneers of a true "science of medicine."

